# PGC-1α regulates alanine metabolism in muscle cells

**DOI:** 10.1371/journal.pone.0190904

**Published:** 2018-01-09

**Authors:** Yukino Hatazawa, Kun Qian, Da-Wei Gong, Yasutomi Kamei

**Affiliations:** 1 Laboratory of Molecular Nutrition, Graduate School of Life and Environmental Science, Kyoto Prefectural University, Kyoto, Japan; 2 Japan Society for the Promotion of Science, Tokyo, Japan; 3 Department of Gastrointestinal Surgery, The First Affiliated Hospital of Chongqing Medical University, Chongqing, China; 4 Department of Medicine, University of Maryland School of Medicine, Baltimore, Maryland, United States of America; Tohoku University, JAPAN

## Abstract

The skeletal muscle is the largest organ in the human body, depositing energy as protein/amino acids, which are degraded in catabolic conditions such as fasting. Alanine is synthesized and secreted from the skeletal muscle that is used as substrates of gluconeogenesis in the liver. During fasting, the expression of PGC-1α, a transcriptional coactivator of nuclear receptors, is increased in the liver and regulates gluconeogenesis. In the present study, we observed increased mRNA expression of PGC-1α and alanine aminotransferase 2 (ALT2) in the skeletal muscle during fasting. In C2C12 myoblast cells overexpressing PGC-1α, ALT2 expression was increased concomitant with an increased alanine level in the cells and medium. In addition, PGC-1α, along with nuclear receptor ERR, dose-dependently enhanced the ALT2 promoter activity in reporter assay using C2C12 cells. In the absence of glucose in the culture medium, mRNA levels of PGC-1α and ALT2 increased. Endogenous PGC-1α knockdown in C2C12 cells reduced ALT2 gene expression level, induced by the no-glucose medium. Taken together, in the skeletal muscle, PGC-1α activates ALT2 gene expression, and alanine production may play roles in adaptation to fasting.

## Introduction

Alanine aminotransferase (ALT, also known as glutamate pyruvate transaminase) is an enzyme catalyzing reversible transamination between pyruvate and glutamate to form alanine and 2-oxoglutarate. By mediating the conversion of these metabolites, ALT plays an important role in gluconeogenesis and amino acid metabolism during fasting [1, 2]. Namely, in the skeletal muscle, ALT transfers the alpha-amino group from glutamate to pyruvate to form alanine. Alanine, released from the skeletal muscle, is used as a substrate for gluconeogenesis in the liver. ALT catalyzes the reaction to form pyruvate from alanine in the liver, which is known as the “glucose-alanine cycle” [1, 2]. There are two ALTs, ALT1 and ALT2, with different tissue distribution. ALT1 expression is relatively high in the colon, intestine, adipose tissue, and liver. ALT2 expression is high in the brain, adipose tissue, liver, and skeletal muscle [1]. The regulation of ALT expression has been examined mainly in the liver. ALT2 expression is increased in the liver in both fasted mice and *ob/ob* diabetic mice [3]. ALT2 expression is increased by glucocorticoid in the liver and hepatocytes [3, 4]. ALT1 expression is increased by fibrates in human hepatocytes [5]. Meanwhile, the regulation of ALT expression in the skeletal muscle remains to be elucidated thus far.

Peroxisome proliferator-activated receptor-gamma coactivator 1-alpha (PGC-1α) is a coactivator of nuclear receptors, such as estrogen receptor-related receptor (ERR) [6, 7]. PGC-1α is expressed in the tissues including the skeletal muscle and liver and is increased by stimulation such as exercise [8] and fasting [9]. PGC-1α in the skeletal muscle is known to promote fatty acid oxidation [6, 7]. Recently, we found that PGC-1α in the skeletal muscle promotes branched chain amino acid (BCAA) metabolism [10–12]. Namely, PGC-1α increases lipid/amino acid usage, instead of glucose, which saves blood glucose usage during fasting. PGC-1α also up-regulates pyruvate dehydrogenase kinase 4 (PDK4) and suppresses the glycolysis pathway in the skeletal muscle, which restricts the use of glucose. In these cases, ERRs and PGC-1α coordinately activate PDK4 and fatty acid oxidation genes [6, 13, 14]. Moreover, PGC-1α promotes gluconeogenesis in the liver, during fasting, by increasing phosphoenolpyruvate carboxykinase (PEPCK) and glucose 6-phosphatase (G6Pase) [9]. Therefore, PGC-1α plays roles during fasting in both the skeletal muscle and liver.

In the present study, we examined whether PGC-1α increases alanine metabolism by regulating ALT in the skeletal muscle.

## Results and discussion

### Gene expression changes of PGC-1α, ALT1, and ALT2 in the liver and skeletal muscle of fasted mice

**Fig 1:**
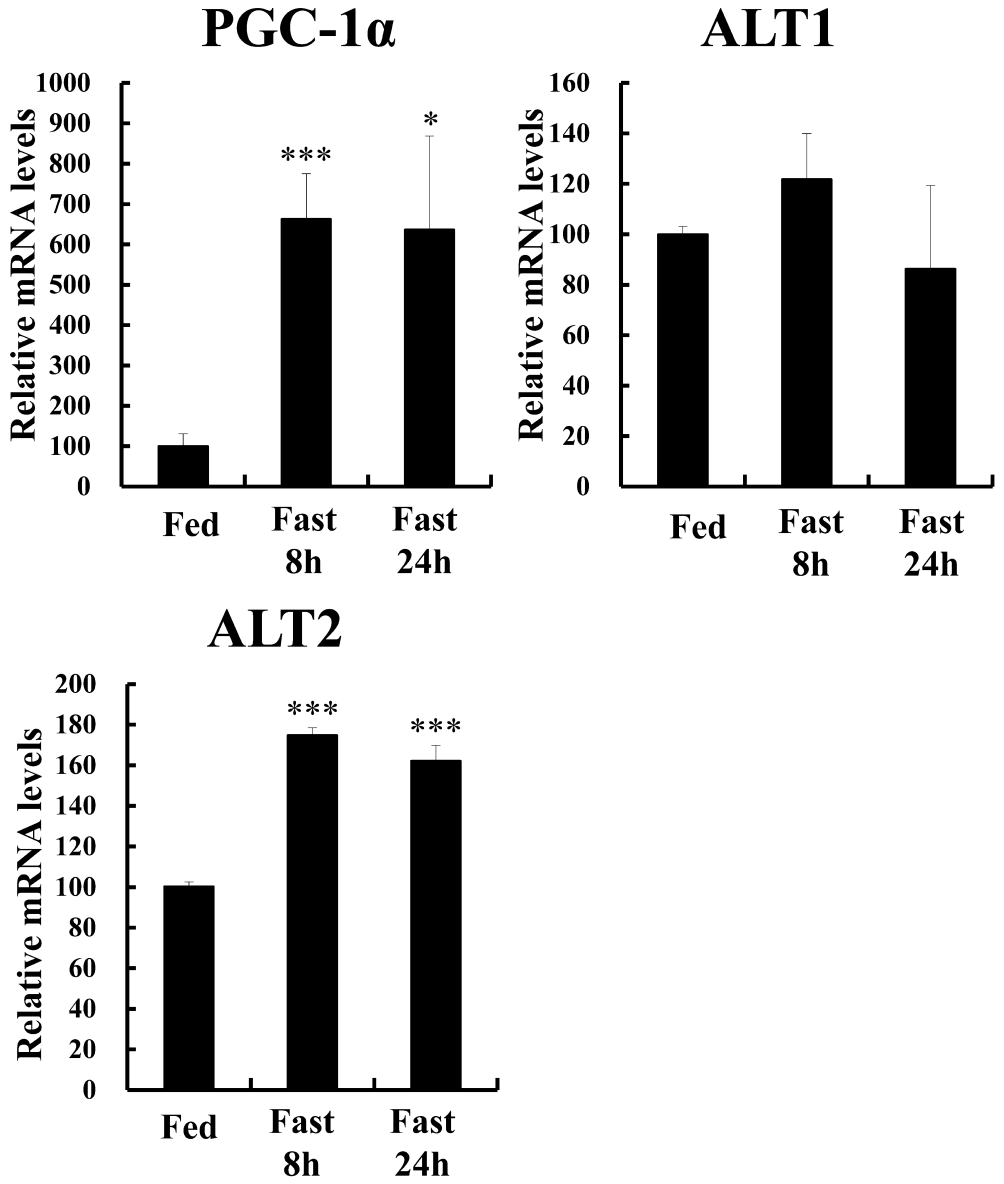
Gene expression in the skeletal muscle of PGC-1α and ALT2, but not ALT1, is increased by fasting. Mice (12-week-old males) were either allowed ad libitum access to food or subjected to fasting for 8 h or 24 h (fed, n = 4; 8 h fasted, n = 4; and 24 h fasted, n = 4). The expression of PGC-1α-b, ALT1, and ALT2 in the skeletal muscle (gastrocnemius) is shown. Quantitative real-time RT-PCR data from fed mice were set at 100 arbitrary units. mRNA levels were normalized to those of 36B4 mRNA. Each value represents mean ± SE (n = 4). ***P < 0.001 and *P < 0.05, relative to fed mice.

We extracted RNA from the mouse liver, skeletal muscle, and kidney after fasting for 8 h and 24 h and examined the gene expression of PGC-1α, ALT1, and ALT2. In the liver, PGC-1α expression was increased by fasting. PEPCK and G6Pase, gluconeogenesis enzymes, known to be increased by PGC-1α [9], were increased by fasting. ALT2, but not ALT1, was increased in the liver by fasting (S1A Fig). Interestingly, in the skeletal muscle (Fig 1) as well as in the liver (S1A Fig), PGC-1α and ALT2 mRNA levels were increased by fasting. Meanwhile, the expression of ALT1 did not change (Fig 1). In the kidney, the gene expression of PGC-1α, ALT1, and ALT2 was not increased by fasting (S1B Fig), suggesting tissue-specific gene regulation. Thus, PGC-1α and ALT2 gene expressions were similarly regulated by fasting in the liver and skeletal muscle. Based on these data, we investigated whether PGC-1α stimulates alanine metabolism via increasing ALT2 expression, in the skeletal muscle.

### Increased expression of ALT2 in C2C12 myoblast cells overexpressing PGC-1α

**Fig 2:**
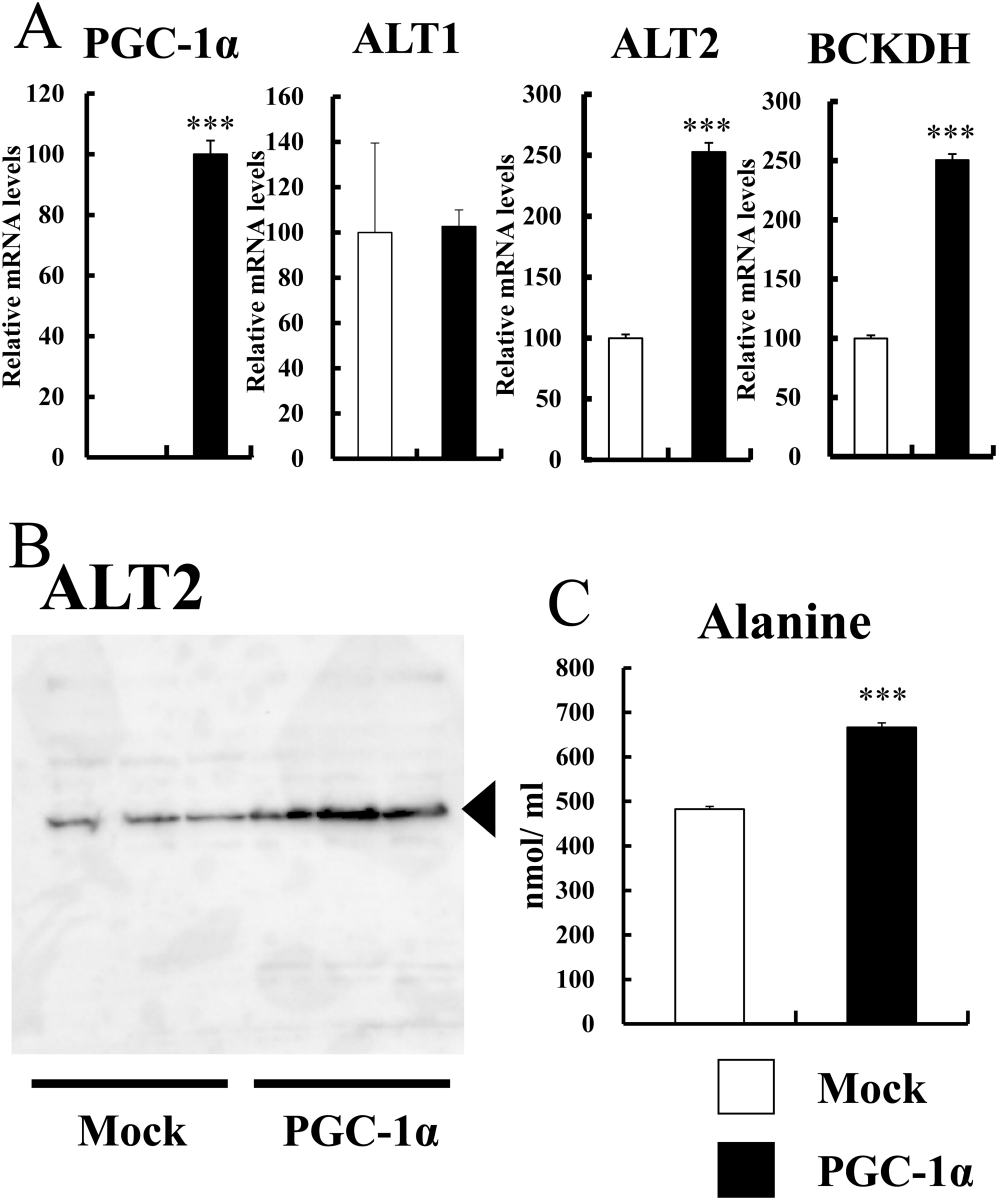
Gene and protein expression of ALT2 and medium alanine level in C2C12 cells overexpressing PGC-1α. A) Gene expression of PGC-1α, ALT1, ALT2 and BCKDH in cultured C2C12 cells overexpressing PGC-1α. Total RNA was isolated from the cells and analyzed by quantitative real-time RT-PCR. Open bars represent mock cells (n = 3), and filled bars represent PGC-1α-overexpressed cells (n = 3). Each value represents mean ± SE (n = 3). The relative values are shown (the mock control is set as 100). For PGC-1α expression, the value was set as 100 in the PGC-1α-overexpressed cells. mRNA levels were normalized to those of 36B4 mRNA. ***P < 0.001. B) Western blot analysis of ALT2 protein in mock cells (control, n = 3) and PGC-1α-overexpressed cells (n = 3). C) Alanine level of the culture medium in C2C12 cells overexpressing PGC-1α. C2C12 cells overexpressing PGC-1α are cultured in DMEM supplemented with 10% FBS until the cells reached confluence. The cells were cultured in DMEM without serum for 48 h, and the culture medium was examined for alanine content. Open bars represent mock cells (n = 3), and filled bars represent PGC-1α-overexpressed cells (n = 3). Each value represents mean ± SE. ***P < 0.001.

To determine the causal relationship between PGC-1α and ALT expression, we examined ALT gene expression in C2C12 myoblast cells overexpressing PGC-1α. Using retrovirus, PGC-1α was stably overexpressed in C2C12 cells. The BCAA metabolism gene expression (BCKDH) was increased by PGC-1α (Fig 2A), which is consistent with our previous study [10], confirming PGC-1α overexpression is functional. The gene expression of ALT2, but not ALT1, was markedly increased in C2C12 cells overexpressing PGC-1α (Fig 2A). Western blot analysis showed an increased protein level of ALT2 in PGC-1α overexpressing cells (Fig 2B). In the PGC-1α overexpressing cells, PGC-1α protein level was not detected, which could be the detection levels of antibodies used were low (data not shown).

### Metabolic pathways changed in C2C12 cells overexpressing PGC-1α

To determine what kind of metabolic pathways are changed in C2C12 cells overexpressing PGC-1α, we performed microarray analysis. More than 2-fold increased genes, compared with control cells, were extracted and used for pathway analysis. As a result, oxidative phosphorylation pathways, tricarboxylic acid cycle, already known to be regulated by PGC-1α, were detected. Moreover, consistent with our previous study, BCAA degradation pathway was detected (S2 Fig). PGC-1α stimulates BCAA degradation in the skeletal muscle [10, 11]. During this process, the amino group is released from BCAA and used for transamination substrate from pyruvate to alanine. Similarly, the alanine-aspartate-glutamate pathway was detected (S2 Fig), suggesting PGC-1α may regulate alanine-related metabolic pathway.

### Alanine level in the medium of C2C12 cells overexpressing PGC-1α

As shown in Fig 2A and 2B, PGC-1α increased ALT2 mRNA and protein levels. Consistently, we previously observed that cellular alanine level increased 1.5-fold in C2C12 cells overexpressing PGC-1α [10]. To examine whether the increased alanine was secreted from the cells, in the present study, we measured alanine level in the medium of C2C12 cells overexpressing PGC-1α. Indeed, alanine level was significantly increased in the medium by PGC-1α overexpression (Fig 2C). This suggests that PGC-1α increases alanine secretion and may affect the outside skeletal muscle tissues.

### Promoter activity of ALT2 gene by increasing the amount of PGC-1α

**Fig 3:**
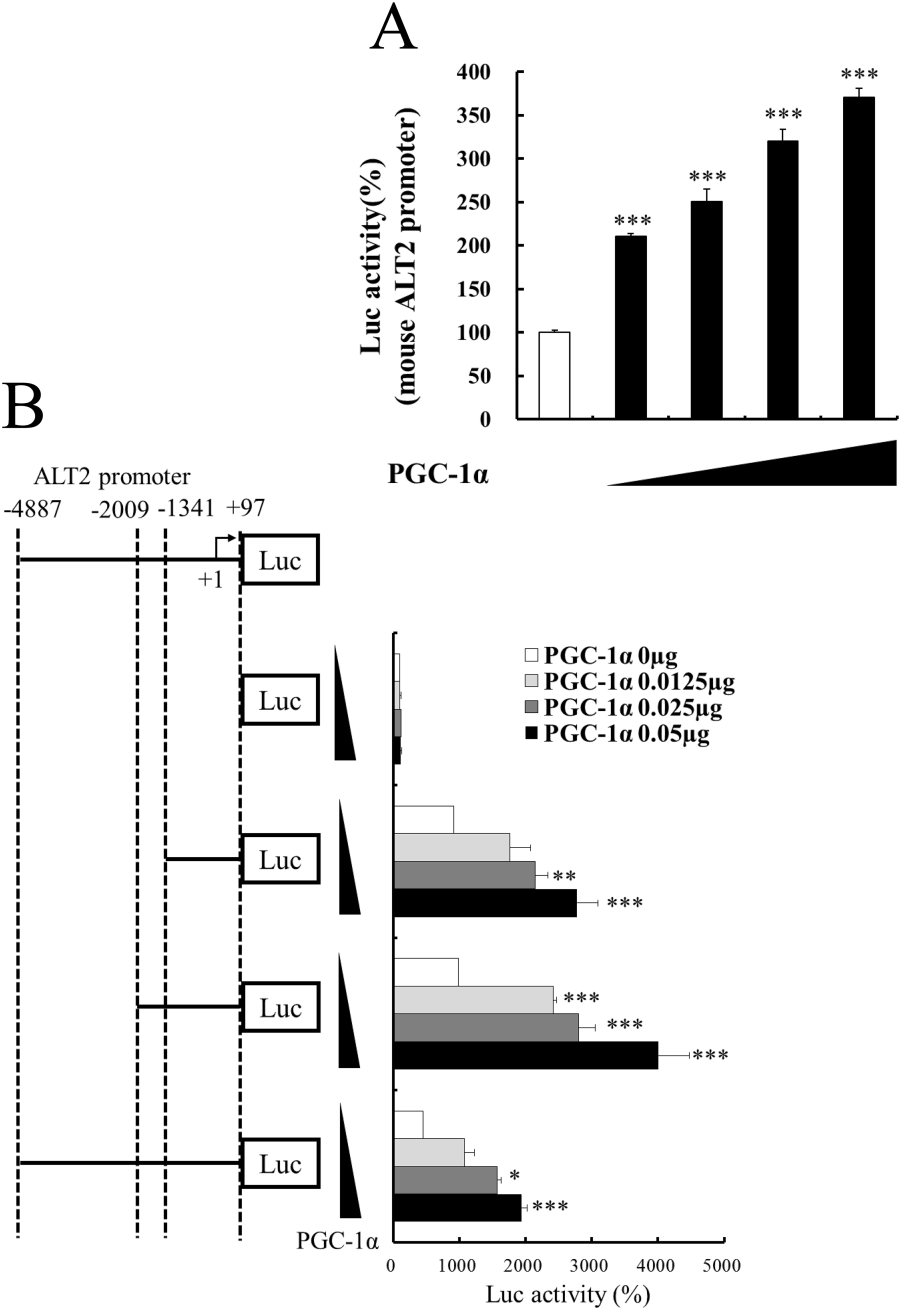
Transient transfection reporter assay of the effect of PGC-1α on the ALT2 promoter. The effect of increasing PGC-1α expression was examined by cotransfection with a reporter plasmid in C2C12 cells. A) The constructs include a 2.0-kb genomic promoter region and the first exon of the ALT2 gene (−2009 to +101, from the transcription start site), the luciferase reporter gene. B) The left panel shows the reporter construct containing ALT2 promoter. Transcription start site (+1) is shown in the panel (arrow). The constructs include 1.4-kb, 2.0-kb, and 4.9-kb genomic promoter regions and the first exon, the luciferase reporter gene. Each value represents mean ± SE (n = 3). ***P < 0.001, **P < 0.01, and *P < 0.05 compared with the samples in the absence of PGC-1α expression vector (open bar).

Because ALT2 gene appeared to be a direct transcriptional target of PGC-1α in C2C12 cells, we tested this hypothesis using a transient transfection reporter assay. We made the constructs to include a 2.0-kb genomic promoter region and the first exon (−2009 to +101, from the transcription start site), the luciferase reporter gene. In the *in vitro* transfection assay, PGC-1α dose-dependently activated the mouse ALT2 reporter construct (Fig 3A and 3B). We also observed an increased luciferase activity using a different length of reporter (−1.4 kb and −4.9 kb), and −2 kb construct showed higher reporter activity by PGC-1α (Fig 3B). This suggests that PGC-1α activates ALT2 gene in the muscle cells.

### Possible transcription factors, involved in the ALT2 gene regulation

Transcriptional factors, which may bind to the ALT2 promoter, are glucocorticoid receptor (GR) and kruppel like factor 15 (KLF15). Alanine level in skeletal muscle of GR-knockout mice was reported to be decreased during fasting [15]. Indeed, we previously showed that PGC-1α interacted with GR [6]. However, dexamethasone (DEX), a synthetic glucocorticoid, treatment did not affect the expression of ALT2 in C2C12 cells (S3 Fig). Meanwhile, in KLF15-knockout mice, gene expression of ALT was reported to be decreased in skeletal muscle [16], suggesting KLF15 mediated regulation of ALT2 expression in skeletal muscle. In our study, however, KLF15 expression vector did not activate, but rather suppressed ALT2-reporter in C2C12 cells (S4 Fig). This suggests that KLF15 may regulate ALT2 expression outside the promoter region that we tested. Thus, GR and KLF15 are not likely to regulate ALT2 gene via PGC-1α in C2C12 cells.

### PGC-1α and ERR additively activated the ALT2 gene promoter

**Fig 4:**
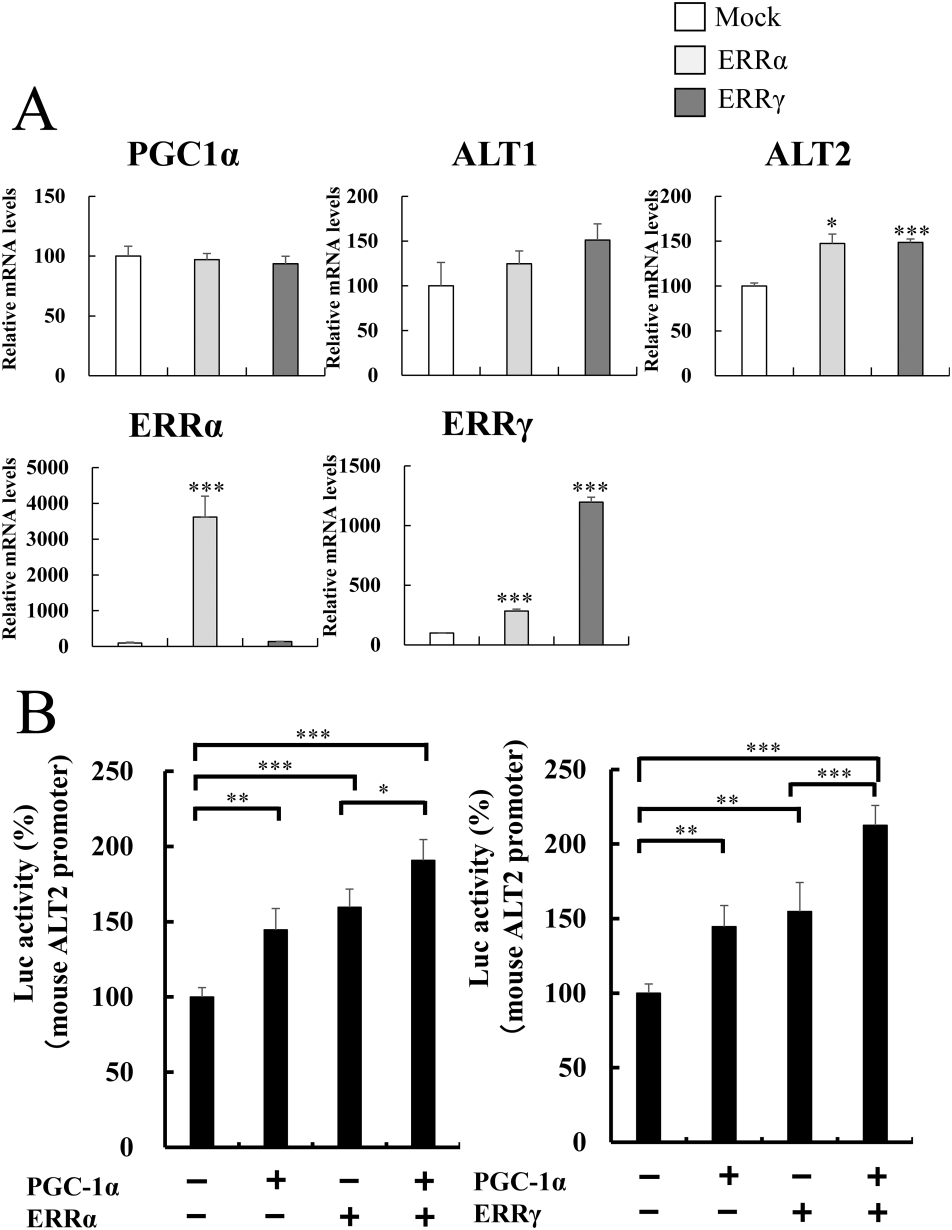
Effect of PGC-1α and ERR on the ALT2 gene. A) Gene expression of PGC-1α, ALT1, ALT2, ERRα and ERRγ in cultured C2C12 cells overexpressing ERRα and ERRγ. Total RNA was isolated from the cells and analyzed by quantitative real-time RT-PCR. Each value represents mean ± SE (n = 3). The relative values are shown (the mock control is set as 100). mRNA levels were normalized to those of 36B4 mRNA. B) The effect of the expression of PGC-1α and ERR (ERRα, left and ERRγ, right) was examined by cotransfection with a reporter plasmid in C2C12 cells. The constructs include a 2.0-kb genomic promoter region and the first exon of the ALT2 gene (−2009 to +101, from the transcription start site), the luciferase reporter gene. Each value represents mean ± SE (n = 3). ***P < 0.001, **P < 0.01, and * P < 0.05.

PGC-1α with ERR activates fatty acid oxidation gene expression and increased the use of lipid, instead of glucose, which saves blood glucose during fasting [6, 13, 14]. ERRα and ERRγ are expressed in the skeletal muscle [6]. Expression level of ERRγ, but not ERRα, was increased by 8 h fasting in the skeletal muscle (S5 Fig). We examined ALT gene expression in C2C12 cells overexpressing ERRα or ERRγ. Interestingly, we observed increased ALT2 (not ALT1) expression in C2C12-ERRα and C2C12-ERRγ cells (Fig 4A). The data suggest that ERR stimulates ALT2 gene expression. Moreover, we examined whether PGC-1α and ERR coordinately activate ALT2 gene expression. In the presence of PGC-1α and ERRα or ERRγ, ALT2 reporter activity was examined. As a result, PGC-1α alone and ERRα or ERRγ alone activated ALT2 reporter. PGC-1α and ERRα or PGC-1α and ERRγ additively increased reporter activity (Fig 4B). Therefore, PGC-1α is likely to activate ALT2 gene expression by mediating ERR. According to the data of Fig 3B, between +1 (transcription start site) and −1.4 kb of the ALT2 promoter, there appears to be responsive elements of transcription factors that are co-activated by PGC-1α. Meanwhile, there are no consensus ERR binding sites (TCAAGGTCA) [17] in the ALT2 promoter region. Thus, there might be ERR binding sites that do not completely match the consensus sequences. This remains to be solved.

### Increased ALT2 expression in no-glucose medium is suppressed by knockdown of PGC-1α in C2C12 cells

**Fig 5:**
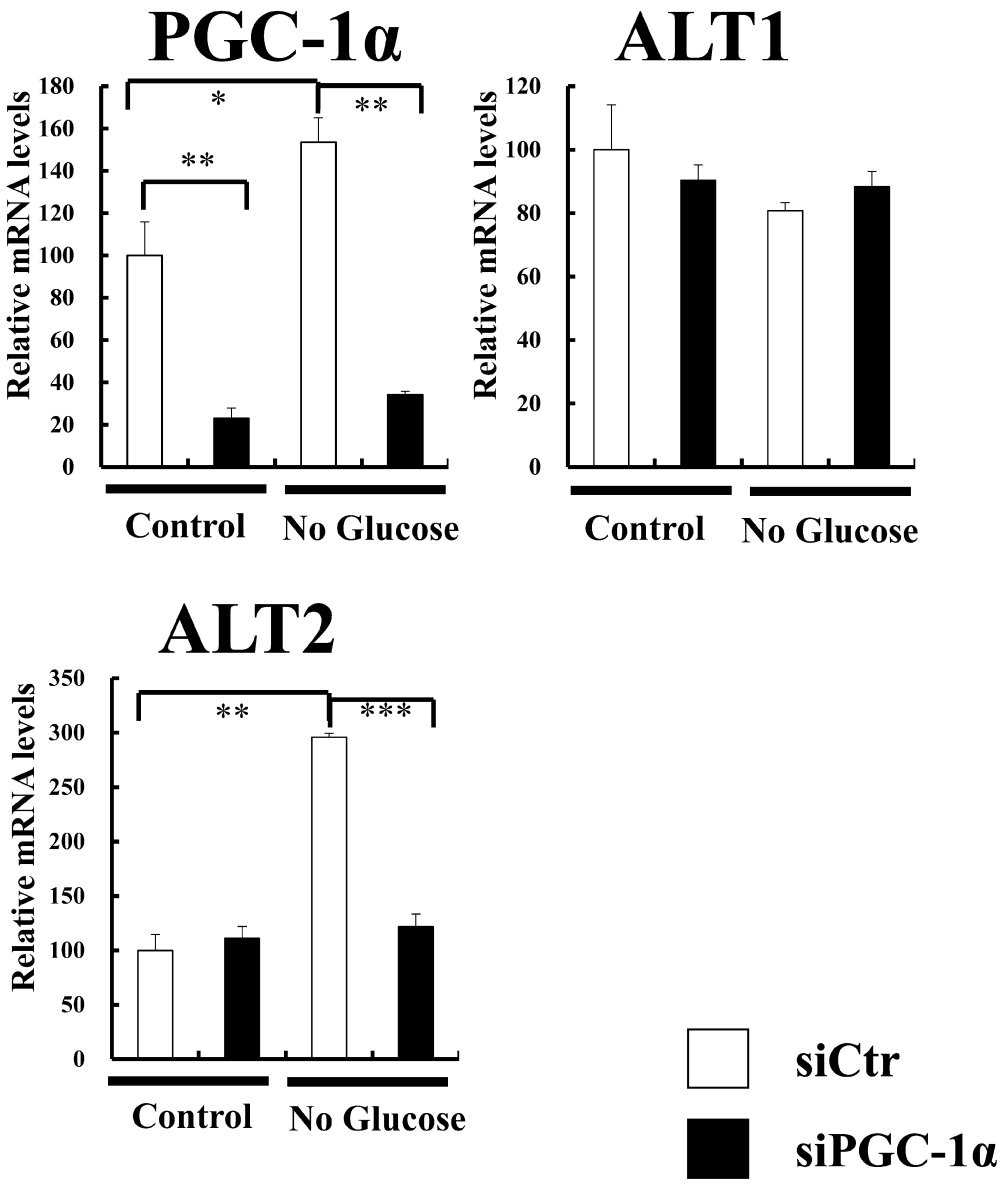
Increased ALT2 expression in no-glucose medium is suppressed by knockdown of PGC-1α in C2C12 cells. C2C12 cells were transfected with control siRNA (open bars) or siRNA of PGC-1α (filled bars) and cultured in control medium (containing 4.5g/L glucose, left) and no-glucose medium (right). After 48 h, total RNA was isolated from the cells and analyzed by quantitative real-time RT-PCR with primers for PGC-1α, ALT1, and ALT2. Endogenous PGC-1α was knocked down by siRNA, and increased ALT2 in no-glucose medium was suppressed by PGC-1α knockdown. Each value represents mean ± SE (n = 3). The relative values are shown (the control; control medium with control siRNA, is set as 100). mRNA levels were normalized to those of 36B4 mRNA. ***P < 0.001, **P < 0.01, and *P < 0.05.

As glucose level decreases during fasting, we next used glucose-free medium (no-glucose medium) to culture C2C12 cells. Interestingly, both PGC-1α and ALT2 expression increased in no-glucose medium (Fig 5). Moreover, increased endogenous PGC-1α in no-glucose medium was knocked down by siRNA of PGC-1α in C2C12 cells, as shown in Fig 5. Interestingly, the gene expression of ALT2, induced by no-glucose medium, was decreased by PGC-1α knockdown (Fig 5). The result further suggests that PGC-1α regulates ALT2 gene expression in the muscle cells. In another catabolic state, we observed that clenbuterol (adrenergic receptor agonist) treatment of C2C12 cells increased endogenous PGC-1α expression concomitant with increased ALT2 expression (S6 Fig). The data also strengthens the observation that endogenous PGC-1α enhances ALT2 expression in C2C12 cells.

## Conclusion

In the present study, we examined the gene expression regulation, regarding alanine synthesis in the skeletal muscle during fasting. Using cultured cells overexpressing and knocked down PGC-1α, we found that PGC-1α enhances ALT2 expression. Our data suggest that alanine synthesis is mediated by PGC-1α and may contribute to the adaptation during fasting.

## Materials and methods

### Animals

C57BL/6J, purchased from Shimizu Laboratory Supplies Co., Ltd., (Kyoto Japan), were maintained at a constant temperature of 24°C with fixed artificial light (12-h light/12-h dark cycle). All animal experiments were performed in accordance with the guidelines of the Kyoto Prefectural University Committee on Animal Research. The protocol was approved by the Committee (No. KPU260407, review board: Dr. Yasuhiro Tsukamoto).

### Quantitative real-time RT-PCR analysis

Gene expression levels were measured as previously described [10]. The primers used are shown in S7 Fig.

### C2C12 cells and cell cultures

C2C12 mouse myoblasts (Riken Cell Bank, Tsukuba, Japan) were cultured in Dulbecco’s modified Eagle’s medium (DMEM) containing 4.5g/L glucose, supplemented with 10% fetal bovine serum (FBS) until the cells reached confluence.

### Stable cell lines

pMX-derived expression plasmid [18] containing PGC-1α cDNA, and pLXSN-derived plasmid (Takara Bio Inc. Shiga, Japan) containing ERRα and ERRγ cDNAs were expressed in C2C12 cells as previously described [18].

### Western blotting analysis

Western blotting was performed as previously described [18] using anti-ALT2 (ab80947, Abcam plc., Tokyo, Japan).

### Amino acid analysis

The culture medium in C2C12 cells overexpressing PGC-1α was examined for alanine content, which was measured by HPLC assays (SRL, Tokyo, Japan).

### cDNA microarray analysis

RNA was isolated from C2C12 cells overexpressing PGC-1α or mock vector. Each sample was labeled and hybridized to the Agilent whole mouse genome (8 × 60K) microarray (Agilent Technologies, Inc., Santa Clara, CA, USA), as previously described [10, 12].

### Functional annotation analysis in genes upregulated by PGC-1α overexpression

We performed pathway analysis using the Kyoto Encyclopedia of Genes and Genomes (KEGG) database resource, as previously described [10].

### Plasmid constructs

As shown in Fig 3B, reporter plasmids were constructed. The constructs included 1.4-kb, 2.0-kb, and 4.9-kb genomic promoter regions of mouse ALT2 gene and the first exon (−1341 to +97, −2009 to +101, and −4887 to +97, from the transcription start site), the luciferase reporter gene.

### Transfection and luciferase assays

C2C12 cells were plated at a density of 1 × 10^5^ cells per well in a 12-well plate in DMEM supplemented with 10% FBS. The luciferase reporter plasmid (0.8 μg), expression plasmid (pCAG-PGC-1α: 0.1, 0.2, 0.4, and 0.8 μg), empty pCAG (up to 0.8 μg), and phRL-TK vector (25 ng: Promega Co., Madison, WI, USA), as an internal control of transfection efficiency, were transfected into C2C12 cells. Expression plasmids for ERRα and ERRγ were as previously described [6]. Expression plasmid of KLF15 (pCMV-mouse KLF15) was purchased from Addgene (Rockvill, MD, USA). The cells were lysed and assayed for luciferase activity using the Dual Luciferase Assay kit (Promega) 24 h after transfection. The activity was calculated as the ratio of firefly luciferase activity to Renilla luciferase activity (internal control) and expressed as an average of triplicate experiments [18].

### Cell culture in no-glucose medium and knockdown of endogenous PGC-1α in C2C12 cells

C2C12 cells were transfected with siRNA of PGC-1α cultured in DMEM, supplemented with 10% FBS. C2C12 cells were changed with glucose-free medium (no-glucose medium) or control medium (DMEM with 10% FBS) and cultured for 24 h. Total RNA was isolated from the cells and analyzed by quantitative real-time RT-PCR.

### Statistical analyses

Statistical comparison was performed by Student’s two-tailed unpaired t-test or one-way analysis of variance followed by Tukey’s post hoc test for more groups. Data were checked for normality and equal variances between groups. P < 0.05 was considered statistically significant, and the significance was marked by ***P < 0.001, **P < 0.01, and *P < 0.05.

## Supporting information

S1 Fig Gene expression of PGC-1α, ALT1, and ALT2 in the liver and kidney, during fasting. Mice (12-week-old males) were either allowed ad libitum access to food or subjected to fasting for 8 h or 24 h (fed, n = 4; 8 h fasted, n = 4; and 24 h fasted, n = 4). A) Expression of PGC-1α, ALT1, ALT2, G6Pase and PEPCK in the liver. B) Expression of PGC-1α, ALT1, and ALT2 in the kidney. Quantitative real-time RT-PCR data from fed mice were set at 100 arbitrary units. mRNA levels were normalized to those of 36B4 mRNA. ***P < 0.001, **P<0.01, and *P <0.05, relative to fed mice.
(PDF)

S2 Fig Pathway analysis. Compared with mock cells, 938 genes were found to be up-regulated (more than 2-fold) in C2C12 cells overexpressing PGC-1α by microarray and classified into KEGG pathway analysis as described in Materials and Methods.
(PDF)

S3 Fig Effect of dexamethasone on the expression level of ALT2 mRNA. C2C12 cells were cultured in DMEM containing 10% FBS and indicated concentration of dexamethasone (DEX) for 2 days. mRNA expression of ALT2 was examined. mRNA levels were normalized to those of 36B4 mRNA. Each value represents mean ± SE (n = 3). The relative values are shown (the control is set as 100, open bar).
(PDF)

S4 Fig Reporter assay of the effect of PGC-1α and KLF15 on the ALT2 promoter. The effect of the expression of PGC-1α and KLF15 was examined by cotransfection with a reporter plasmid in C2C12 cells. The constructs included a 2.0-kb genomic promoter region and the first exon of the ALT2 gene (−2009 to +101, from the transcription start site), the luciferase reporter gene. Each value represents mean ± SE (n = 3). ***P < 0.001.
(PDF)

S5 Fig Gene expression of ERRα and ERRγ in the skeletal muscle, during fasting. Mice (12-week-old males) were either allowed ad libitum access to food or subjected to fasting for 8 h or 24 h (fed, n = 4; 8 h fasted, n = 4; and 24 h fasted, n = 4). Expression of ERRα and ERRγ in the skeletal muscle is shown. Quantitative real-time RT-PCR data from fed mice were set at 100 arbitrary units. mRNA levels were normalized to those of 36B4 mRNA. **P<0.01, relative to fed mice.
(PDF)

S6 Fig Effect of clenbuterol on the expression level of PGC-1α and ALT2 mRNA. C2C12 cells were cultured in DMEM containing 2% FBS and 100nM of clenbuterol (clen) for 8 hours. mRNA expression of PGC-1α, ALT1 and ALT2 was examined. mRNA levels were normalized to those of 36B4 mRNA. Each value represents mean ± SE (n = 3). The relative values are shown (the control is set as 100, open bar). *P < 0.05.
(PDF)

S7 Fig List of primers for quantitative real-time RT-PCR. (PDF)
