# Supplementary figures and images for: PGC-1α regulates alanine metabolism in muscle cells
Source: PLoS One. 2018 Jan 9;13(1):e0190904. doi: 10.1371/journal.pone.0190904 (PMC5760032; doi:10.1371/journal.pone.0190904)

# S1 Fig

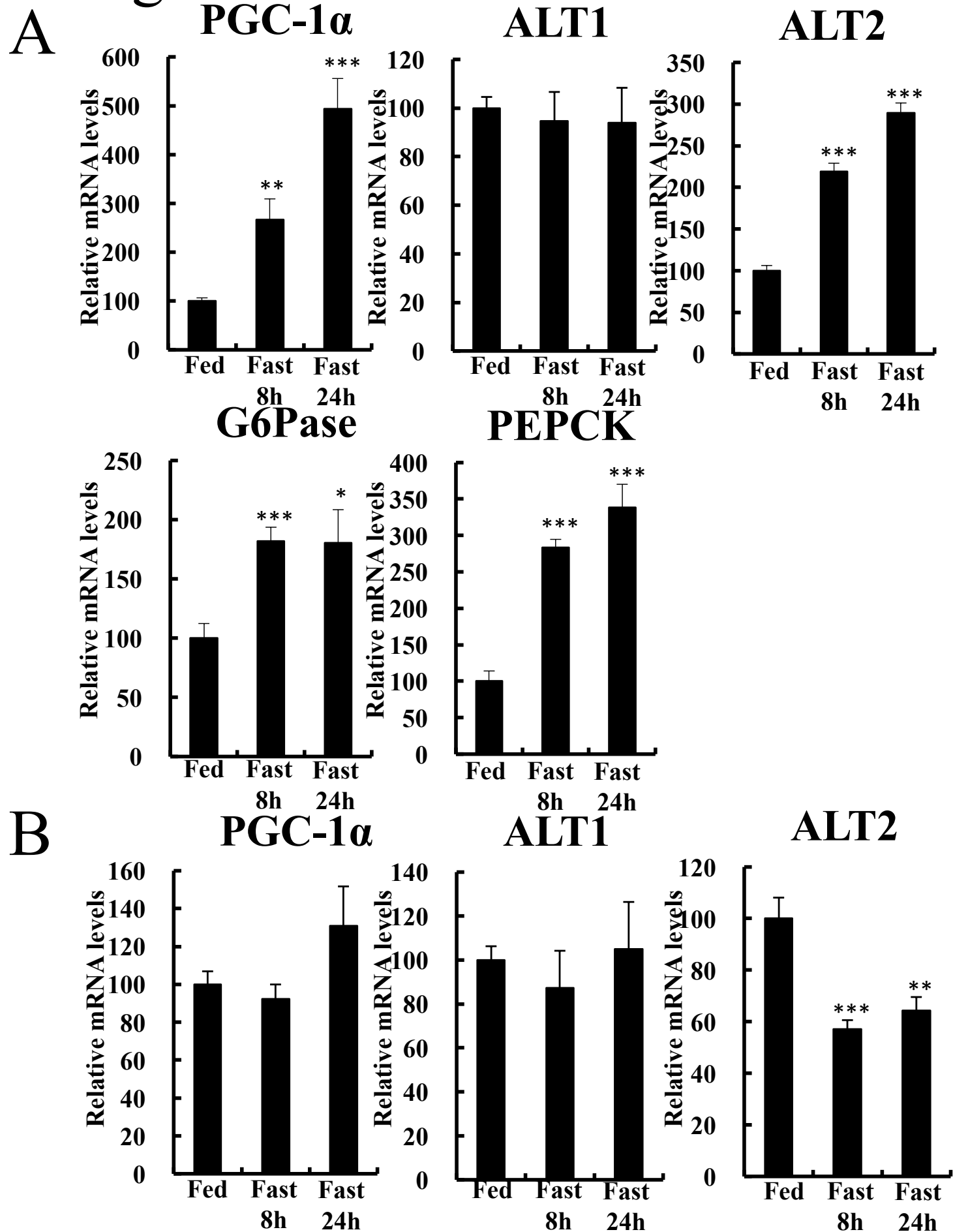

Supplement: S1 Fig — Mice (12-week-old males) were either allowed ad libitum access to food or subjected to fasting for 8 h or 24 h (fed, n = 4; 8 h fasted, n = 4; and 24 h fasted, n = 4). A) Expression of PGC-1α, ALT1, ALT2, G6Pase and PEPCK in the liver. B) Expression of PGC-1α, ALT1, and ALT2 in the kidney. Quantitative real-time RT-PCR data from fed mice were set at 100 arbitrary units. mRNA levels were normalized to those of 36B4 mRNA. ***P < 0.001, **P<0.01, and *P <0.05, relative to fed mice. (PDF) [file pone.0190904.s001.pdf]

# S3 Fig

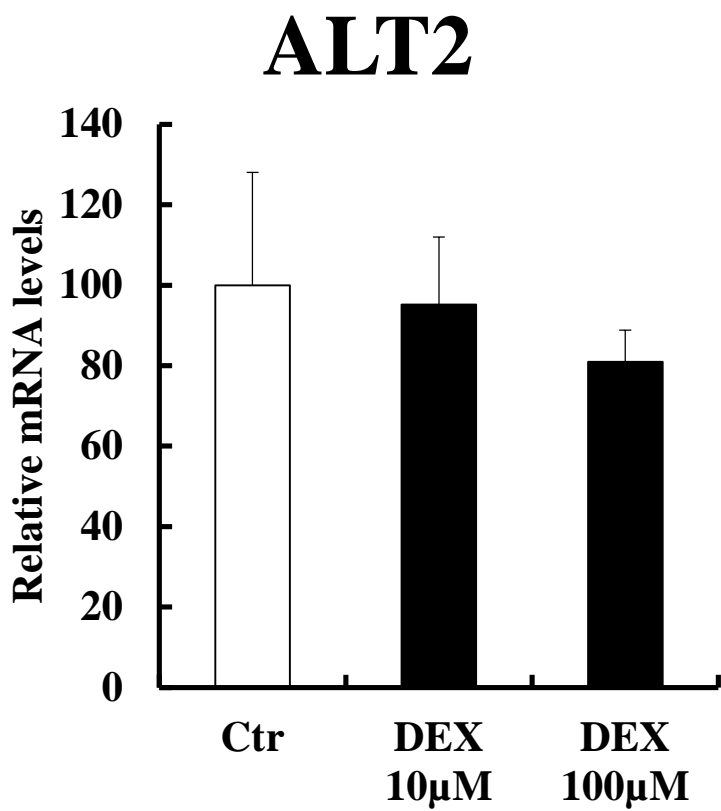

Supplement: S3 Fig — C2C12 cells were cultured in DMEM containing 10% FBS and indicated concentration of dexamethasone (DEX) for 2 days. mRNA expression of ALT2 was examined. mRNA levels were normalized to those of 36B4 mRNA. Each value represents mean ± SE (n = 3). The relative values are shown (the control is set as 100, open bar). (PDF) [file pone.0190904.s003.pdf]

S4 Fig

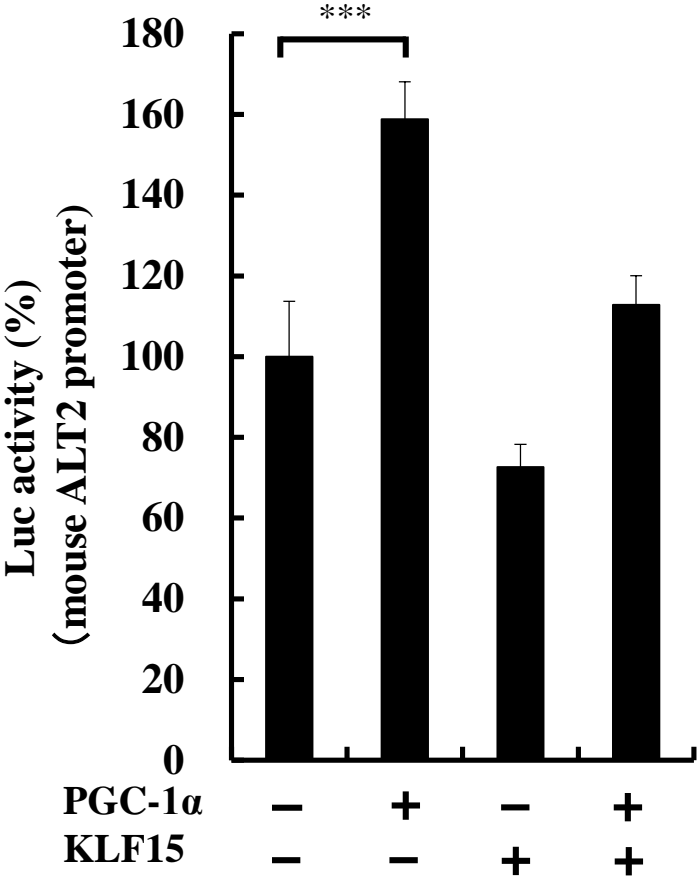

Supplement: S4 Fig — The effect of the expression of PGC-1α and KLF15 was examined by cotransfection with a reporter plasmid in C2C12 cells. The constructs included a 2.0-kb genomic promoter region and the first exon of the ALT2 gene (−2009 to +101, from the transcription start site), the luciferase reporter gene. Each value represents mean ± SE (n = 3). ***P < 0.001. (PDF) [file pone.0190904.s004.pdf]

# S5 Fig

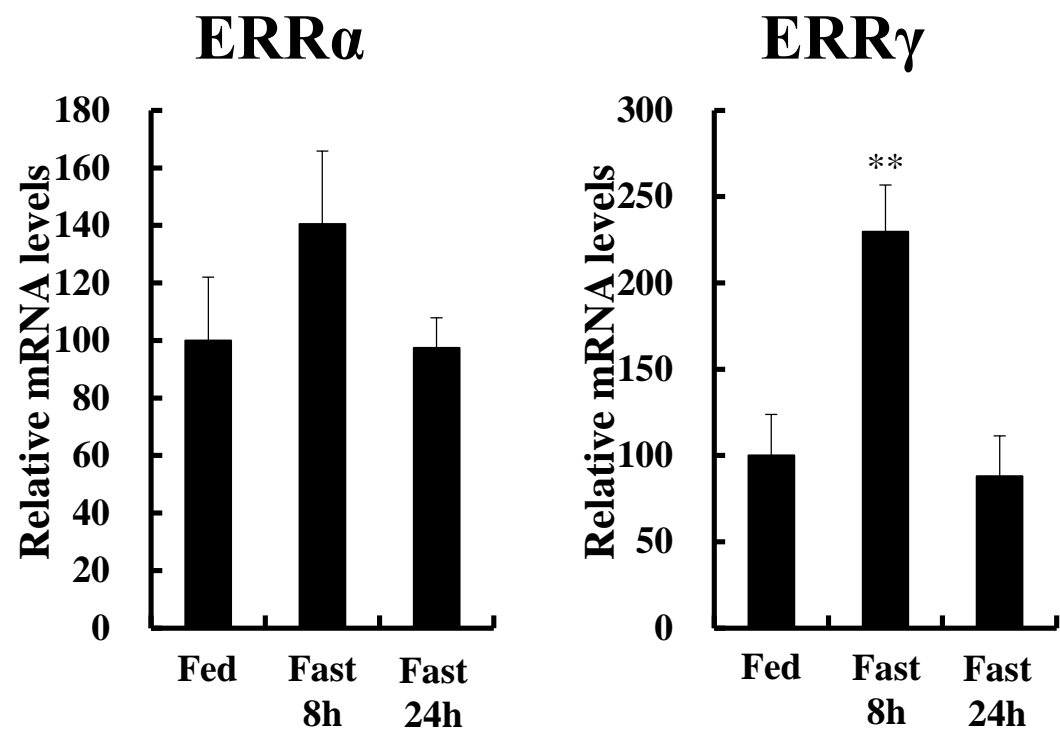

Supplement: S5 Fig — Mice (12-week-old males) were either allowed ad libitum access to food or subjected to fasting for 8 h or 24 h (fed, n = 4; 8 h fasted, n = 4; and 24 h fasted, n = 4). Expression of ERRα and ERRγ in the skeletal muscle is shown. Quantitative real-time RT-PCR data from fed mice were set at 100 arbitrary units. mRNA levels were normalized to those of 36B4 mRNA. **P<0.01, relative to fed mice. (PDF) [file pone.0190904.s005.pdf]

S6 Fig

**PGC1 $\alpha$**

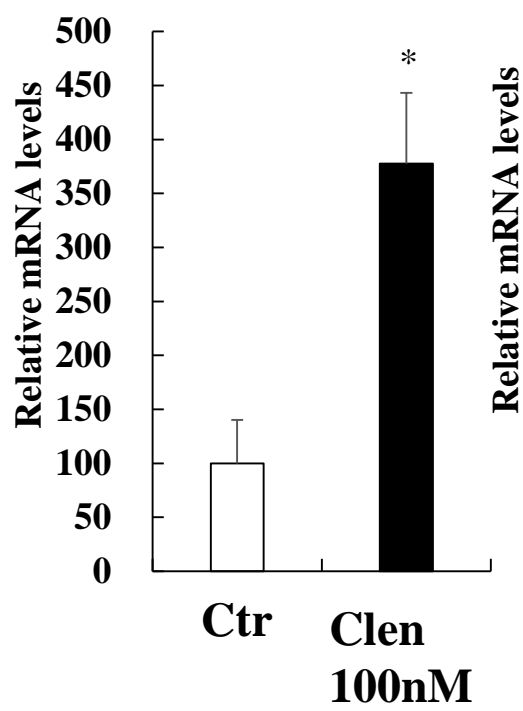

**ALT1**

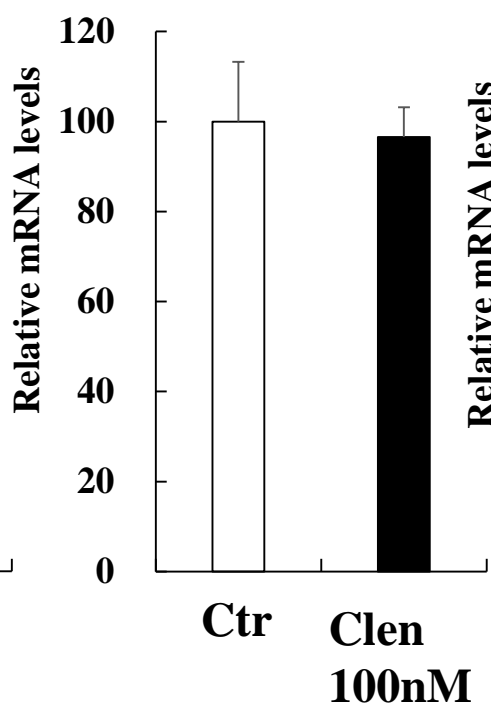

**ALT2**

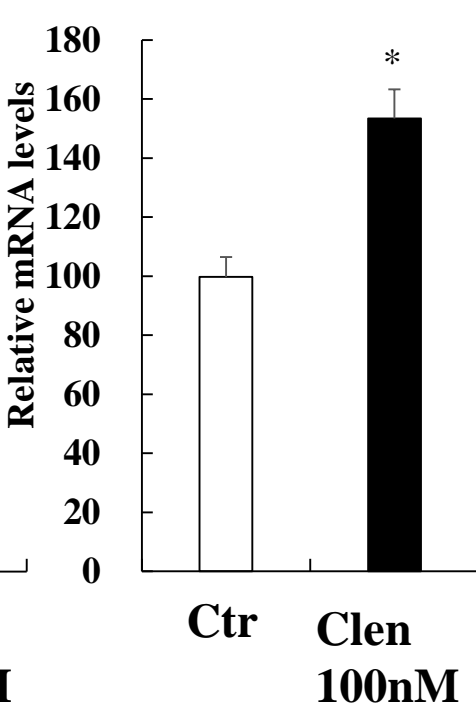

Supplement: S6 Fig — C2C12 cells were cultured in DMEM containing 2% FBS and 100nM of clenbuterol (clen) for 8 hours. mRNA expression of PGC-1α, ALT1 and ALT2 was examined. mRNA levels were normalized to those of 36B4 mRNA. Each value represents mean ± SE (n = 3). The relative values are shown (the control is set as 100, open bar). *P < 0.05. (PDF) [file pone.0190904.s006.pdf]
