# Supplementary material for: PGC-1α regulates alanine metabolism in muscle cells
Source: PLoS One. 2018 Jan 9;13(1):e0190904. doi: 10.1371/journal.pone.0190904 (PMC5760032; doi:10.1371/journal.pone.0190904)
Supplement: S2 Fig — Compared with mock cells, 938 genes were found to be up-regulated (more than 2-fold) in C2C12 cells overexpressing PGC-1α by microarray and classified into KEGG pathway analysis as described in Materials and Methods. (PDF) [file pone.0190904.s002.pdf]

S2 Fig

Pathway	P-Value	Benjamini
Oxidative phosphorylation	1.8E-10	2.4E-08
Citrate cycle (TCA cycle)	6.8E-10	4.6E-08
Parkinson's disease	1.2E-08	5.2E-07
Cardiac muscle contraction	4.3E-07	1.5E-05
Huntington's disease	0.000002	5.3E-05
Pyruvate metabolism	2.6E-06	5.7E-05
Alzheimer's disease	0.000029	5.6E-04
Glyoxylate and dicarboxylate metabolism	0.011	1.7E-01
Propanoate metabolism	0.012	1.6E-01
p53 signaling pathway	0.017	2.1E-01
Arginine and proline metabolism	0.021	2.3E-01
Synthesis and degradation of ketone bodies	0.035	3.3E-01
Apoptosis	0.047	3.9E-01
Valine, leucine and isoleucine degradation	0.049	3.8E-01
Glycolysis / Gluconeogenesis	0.053	3.9E-01
Alanine, aspartate and glutamate metabolism	0.06	4.1E-01
Melanoma	0.062	4.0E-01
Pyrimidine metabolism	0.069	4.1E-01
Glycine, serine and threonine metabolism	0.071	4.1E-01
Cysteine and methionine metabolism	0.076	4.1E-01
Inositol phosphate metabolism	0.079	4.1E-01
Butanoate metabolism	0.099	4.7E-01
