# Supplementary material for: PGC-1α regulates alanine metabolism in muscle cells
Source: PLoS One. 2018 Jan 9;13(1):e0190904. doi: 10.1371/journal.pone.0190904 (PMC5760032; doi:10.1371/journal.pone.0190904)
Supplement: S7 Fig — (PDF) [file pone.0190904.s007.pdf]

S7 Fig

Gene	Forward	Reverse
36B4	GGCCCTGCACTCTCGCTTTC	TGCCAGGACGCGCTTGT
PGC1α	CGGAAATCATATCCAACCAG	TGAGGACCGCTAGCAAGTTTG
PGC1α-b	GACATGGATGTTGGGATTGTCA	ACCAACCAGAGCAGCACATTT
ALT1	GCGCCAGGGTGTGAAGAA	GCTTGTGCATCCCCAATATTG
ALT2	GAAGGAAGTAGCCGCATCCA	AGGAAAAGCT GTAGACCGTCACA
BCKDH	CGGCAACGATGTGTTTGCTG	ATTGACCTCGTCCACCGAAC
PEPCK	GGAAGGACAAAGATGGCAAGTTC	AGGCGTTTTCCTTAGGGATGTAG
G6Pase	TCGTGGCTGGAGTCTTGTCA	GGCTGGCAAAGGGTGTAGTG
ERRα	GCAAAGCCTTCTTCAAGAGGAC	GGAGGCCGGACAGCTGT
ERRγ	CATTGGATGGGCAAAACATATTC	TCTGCCAGGGACAGTGTGG
